# Evaluation of the post-processing algorithms SimGrid and S-Enhance for paediatric intensive care patients and neonates

**DOI:** 10.1007/s00247-021-05279-2

**Published:** 2022-02-22

**Authors:** Paul-Christian Krueger, Katharina Ebeling, Matthias Waginger, Katja Glutig, Marcel Scheithauer, Peter Schlattmann, Hans Proquitté, Hans-Joachim Mentzel

**Affiliations:** 1 Section of Pediatric Radiology, Department of Diagnostic and Interventional Radiology, University Hospital Jena, Jena, Germany; 2 Department of Diagnostic and Interventional Radiology, University Hospital Jena, Jena, Germany; 3 Center for Clinical Studies, University Hospital Jena, Jena, Germany; 4 Section of Neonatology and Pediatric Intensive Care Medicine, Department of Pediatrics, University Hospital Jena, Jena, Germany

**Keywords:** Deep learning, Digital radiography, Image enhancement, Neonatal intensive care unit, Pediatric intensive care unit, Radiation dosage, Radiography

## Abstract

**Background:**

Post-processing software can be used in digital radiography to achieve higher image quality, especially in cases of scattered radiation. SimGrid is a grid-like software based on a Convolutional Neuronal Network that estimates the distribution and degree of scattered radiation in radiographs and thus improves image quality by simulating an anti-scatter grid. S-Enhance is an algorithm programmed to improve contrast visibility of foreign material.

**Objective:**

The objective of this study was to evaluate the SimGrid and S-Enhance digital radiography post-processing methods for neonatology and paediatric intensive care.

**Materials and Methods:**

Two hundred and ten radiographs from the neonatal (*n* = 101, 0 to 6 months of age) and paediatric (*n* = 109, 6 months to 18 years of age) intensive care units performed in daily clinical routine using a mobile digital radiography system were post-processed with one of the algorithms, anonymized and then evaluated comparatively by two experienced paediatric radiologists. For every radiograph, patient data and exposure data were collected and analysed.

**Results:**

Analysis of different radiographs showed that SimGrid significantly improves image quality for patients with a weight above 10 kg (range: 10–30 kg: odds ratio [OR] = 6.683, *P* < 0.0001), especially regarding the tracheobronchial system, intestinal gas, and bones. Utilizing S-Enhance significantly advances the assessment of foreign material (OR = 136.111, *P* < 0.0001) and bones (OR = 34.917, *P* < 0.0001) for children of all ages and weight, whereas overall image quality decreases.

**Conclusion:**

SimGrid offers a differentiated spectrum in image improvement for children beyond the neonatal period whereas S-Enhance especially improves visibility of foreign material and bones for all patients.

## Introduction

The development of digital radiography, which enables the use of post-processing algorithms, is important progress, especially in paediatric radiology. The overall objective of such algorithms is to improve image quality for diagnostic interpretation, and to reduce radiation dose to the patient [1]. Image quality may be decreased by scattered radiation that arises from x-rays deflected while passing the human body [2]. Grids are used to reduce scattered radiation and image blurring, but they require a higher radiation dose because unscattered radiation relevant for the final image is blocked as well [3].

**Fig. 1:**
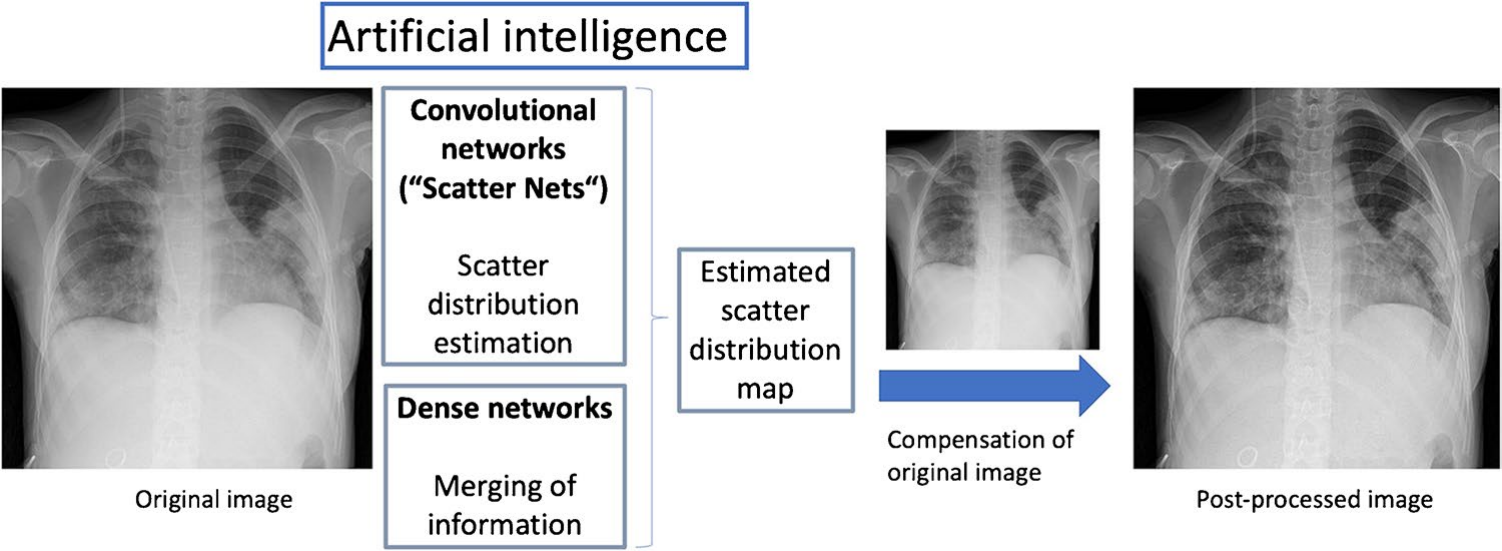
A block diagram illustrates the image processing with SimGrid [5] in an 8-year-old boy (weight: 25 kg) with pneumonia. The original image (near right) after post-processing with convolutional neural networks shows an estimated scatter distribution map resulting in the post-processed image (far right) with improved assessment of lung structures as well as improved contrast

**Fig. 2:**
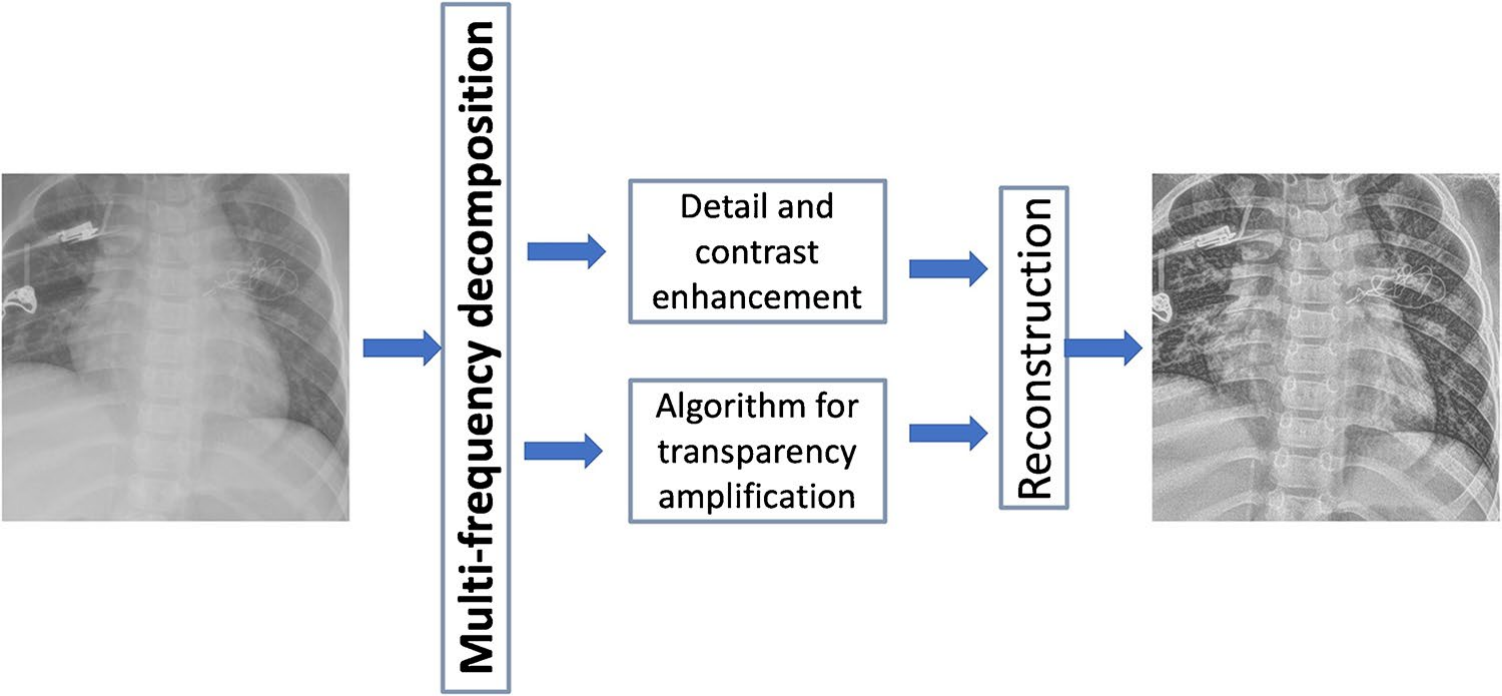
A block diagram illustrates the image processing with S-Enhance [23] in a 2-year-old girl (weight: 11 kg) with postinterventional foreign material in projection to the left part of the chest. The original image (near right) is composed in two different ways with detail and contrast enhancement as well as an algorithm for transparency amplification resulting in the post-processed image (far right)

SimGrid is a deep-learning algorithm based on ScatterNets, which is a Convolutional Neuronal Network (CNN) that estimates scattered radiation and has been pretrained and optimized on more than 30.000 images [4]. The raw input image is compensated with a map of the estimated scattered radiation, resulting in an image similar to an image with an anti-scatter grid, which makes the software flexible to various exposure parameters (Fig. 1) [5]. S-Enhance is software that optimizes detail detection in tubes and lines as well as contrast control by enhancing visual recognition of tubes and inserted lines (Fig. 2) [6]. So far, there are studies on body phantoms and adults that suggest a positive effect from SimGrid on image quality and a possible dose reduction for adults [4, 7]. There are limited data in children and neonates. An important difference compared to adults is the use of anti-scatter grids only for a patient diameter more than 12 – 15 cm [8]. As SimGrid practically works as a replacement for an anti-scatter grid, it would be interesting to see if it still has a positive effect for smaller patients where a grid is not used normally. If a positive effect could be shown, a dose reduction might also be possible. This is very important, especially for children, as some of their organs are very sensitive to radiation due to body growth and metabolic turnover, which includes high mitosis rates and the immaturity of organs and repair mechanisms in preterm infants [9]. Their body proportions are different from those of adults causing more radiation exposition for different organs despite collimation [10]. Children also have more years ahead of them, making malignant tumours through radiation, as well as transmission of genetic aberrations to further generations more probable [11–14]. Despite that, diagnostic imaging with x-ray is essential in this age group to diagnose diseases, which are often difficult to differentiate in very young patients and especially in postoperative controls [15]. That makes dose reduction a huge and important goal of paediatric radiology, to which post-processing algorithms have the potential to contribute.

## Materials and methods

This retrospective study was approved by the local ethics committee (2018–1185-data).

### Patients

The study contained 210 radiographs of 134 children (age range: 0–18 years, mean: 4.22 years) having routine care imaging (with justified indications) in a university hospital between July 2017 and August 2018. For further evaluation, patient data including age, weight, gender, week of pregnancy at birth and medical issues were recorded.

### Image acquisition

All radiographs were created on a mobile digital radiography system (GM85; Samsung Electronics Co. Ltd., Suwon, Korea) under current guidelines for paediatric imaging in anteroposterior projection without a grid. Paediatric standard filtration with 1-mm aluminium (Al) and 0.1-mm copper (Cu) was used.

For further analysis, the parameters current voltage (kV), current–time product (mAs), exposure time (ET), dose area product (DAP), field of view (FoV) and exposure index (EI) were captured.

### Post-processing

First, all images were post-processed using standard software S-Vue (Samsung Electronics Co. Ltd., Suwon, Korea) to optimize image sharpness and clarity. Later, all radiographs were additionally post-processed with SimGrid and S-Enhance separately for further evaluation.

### Image evaluation

All radiographs were evaluated retrospectively by two experienced paediatric radiologists (reader one [H.J.M.] with more than 20 years and reader two [P.C.K.] with more than 5 years of experience in paediatric imaging) by comparing the original image with the processed ones using a 3-point Likert scale (0 = same quality, 1 = image without SimGrid/S-Enhance superior, 2 = image with SimGrid/S-Enhance superior). The radiologists were blinded for patient data and which of the x-ray images was changed with the algorithm. Before evaluation, both readers practiced image assessment on five cases. Further assessment was done independently.

In accordance with guidelines of the German Federal Association for quality assurance in x-ray imaging diagnostics, the following parameters were evaluated for chest radiographs for SimGrid and S-Enhance separately and in case of an abdominal radiograph only for SimGrid: clear visualisation of bones (spine, ribs), trachea, central, peripheral and retrocardiac vessels as well as foreign material and indwelling support devices. Overall image noise, especially for abdominal imaging visualisation of intestinal gas, contrast media effect as well as overall image quality and interpretability relevant to the clinical question were evaluated.

### Statistical analysis

For statistical analysis, the 3-point Likert scale was reduced to 2 points (0 = post-processed image not better or same quality and 1 = post-processed image better). A general estimating equation (GEE) model using SPSS 25.0 was performed to assess the strength of the association between different parameters and image improvement through the technologies expressed as an odds ratio (OR). *P* < 0.05 was considered significant. The influence of the second reviewer was analysed as a covariate and the odds ratio determined the level of difference in assessment of the two readers.

## Results

### Patients

One hundred and nine radiographs were acquired on the paediatric intensive care unit (48 male; age range: 6 months to 18 years, mean: 6.4 years, median: 3.8 years) and 101 radiographs originated from the neonatal intensive care unit (42 male; age range: 0 to 6 months, mean: 3 weeks, median: 2 days).

Acquired images included 130 chest, 31 abdomen and 49 combined chest and abdomen. Radiography was indicated in cases of respiratory distress syndrome, infection, acute abdominal emergency, syndrome screening, trauma and postinterventional imaging.

Patients were grouped by age and weight for further analysis (Table 1).

**Table 1:** Summary of patient data grouped by age and weight

Patient age in years	Number of patients
< 0.5	62
0.5– < 1	10
1–4	35
5–9	11
10–18	16
Patient weight in Kg	Number of patients
< 1	19
1–4	40
5–9	18
10–29	39
30–49	6
50–69	10
70–90	2

*Kg* kilogram

### Image evaluation for SimGrid

Fifty-six percent (*n* = 75) of the 134 images were rated as “with SimGrid superior,” 41% (*n* = 55) were rated as “same quality” and 3% (*n* = 4) were rated as “without SimGrid superior.”

A similar picture arises for assessment of different anatomical regions with 55% better visualisation for chest (42% same and 3% inferior), 56% for abdomen (42% same and 2% inferior) and 59% for combination of chest and abdomen (35% same and 6% inferior), respectively using SimGrid.

### Influencing factors

There was a significant improvement of ratings for patients from 1 to 5 years of age (OR = 4.48, *P* < 0.05) while for patients younger than 6 months (OR = 0.46, *P* < 0.05) there was significantly no improvement and between 6 months and 1 year no significant improvement (OR 0.86, *P* = 0.85). For the age group of 5 to 10 years (OR = 8.35, *P* < 0.05) and older than 10 years (OR 67.24, *P* < 0.05), a significant improvement of ratings was recorded.

**Fig. 3:**
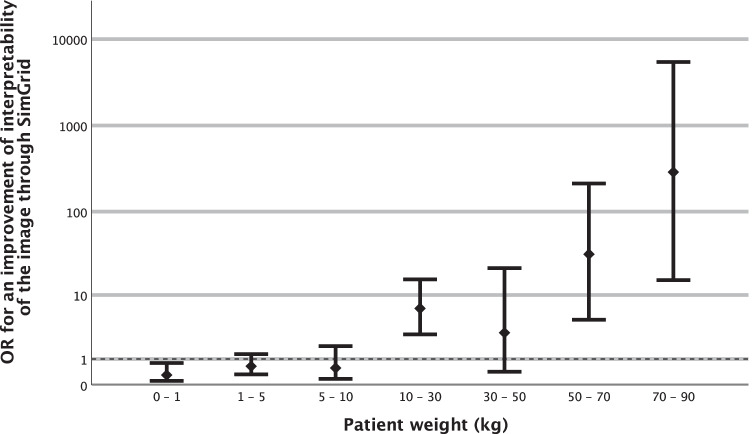
A bar chart shows the odds ratio (OR) for improved interpretability of the images through SimGrid influenced by patient weight, reference line for OR > 1 (improvement)

**Fig. 4:**
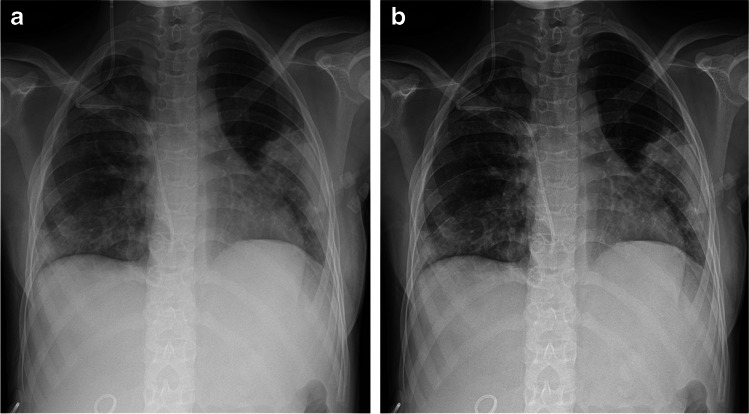
An anteroposterior chest radiograph (69.6 kV, 4.0 mAs, 4.51 cGy·cm^2^, exposure index: 345.50), unprocessed image (**a**) versus a SimGrid image (**b**) in an 8-year-old boy (weight: 25 kg) with pneumonia

SimGrid significantly improved images for patients with a weight of 10 kg or more (OR = 8.31, *P* < 0.05) (Figs. 3 and 4). For neonates with very low weight, there significantly no improvement (OR for < 1 kg = 0.27, *P* < 0.05). For newborns with a weight between 1–5 kg and young children with a weight between 5–10 kg, there was no significant improvement (OR for 1–5 kg = 0.65, *P* = 0.21; OR for 5–10 kg = 0.57, *P* = 0.34).

**Fig. 5:**
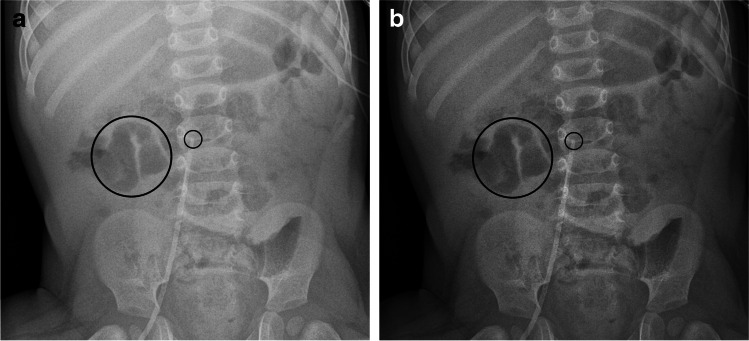
An anteroposterior abdominal radiograph (59.8 kV, 2.0 mAs, 0.57 cGy·cm^2^), unprocessed image (**a**) versus a SimGrid image (**b**) in a 1-year-old boy with central venous catheter from right femoral vein with the tip in the inferior vena cava (*small black ring*) and intestinal gas in the small bowel (*large black ring*)

**Fig. 6:**
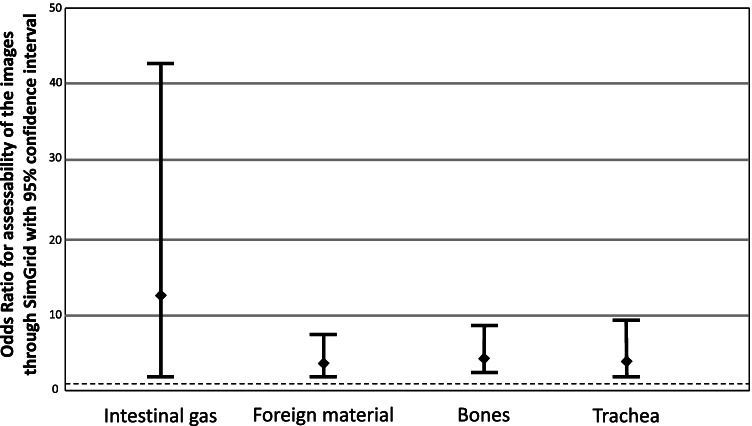
Results differentiating between different evaluation parameters for SimGrid with a red line marking the odds ratio (OR) = 1

For various exposure conditions, evaluation showed different results. Results were best for kV-values above 70 kV (OR = 33.79, *P* < 0.05), mAs-values between 2 and 3 mAs (OR = 58.14, *P* < 0.05), higher dose products (cGy/cm^2^) (e.g., above 5: OR = 21.05, *P* < 0.05) and exposure indices (EI) over 500 (OR = 13.44, *P* < 0.014). Improvement of assessability was seen especially for intestinal gas (OR = 8.27, *P* = 0.010) (Fig. 5), foreign material (OR = 3.19, *P* = 0.001) and bones (OR = 4.18, *P* < 0.0001) (Fig. 6). Results for all parameters are demonstrated in Table 2.

**Table 2:** Odds ratio (OR) for interpretability of the radiographs using SimGrid for the different radiographic evaluation parameters

SimGrid			95% confidence interval for OR		
Parameters	*n*	OR	Lower bound	Upper bound	*P*-value
Bones	134	4.18	2.10	8.32	**<0.001**
Trachea	103	4.12	1.77	9.59	**0.001**
Central vessels	103	0.89	0.41	1.94	0.77
Peripheral vessels	103	0.24	0.11	0.55	**0.001**
Retrocardiac vessels	103	1.31	0.61	2.83	0.49
Foreign material	131	3.19	1.58	6.47	**0.001**
Image noise	134	1.43	0.73	2.81	0.30
Psoas	31	0.08	0.02	0.46	**0.004**
Intestinal gas	31	8.27	1.65	41.31	**0.01**
Contrast media effect	7	0.56	0.03	11.23	0.71
Overall quality	134	1.82	0.93	3.57	0.08
Quality regarding the clinical question	134	1.82	0.92	3.60	0.08

*n* number of evaluations

Bold indicates statistical significance (*P* < 0.05)

### Image evaluation for S-Enhance

Sixty-one percent (*n* = 47) of the 76 images were rated as “with S-Enhance superior,” 14% (*n* = 9) were rated as “same quality” and 25% (*n* = 20) were rated as “without S-Enhance superior.” Assessment of foreign material and visualisation of bone structures were especially improved (92% and 86%, respectively). On the other hand, overall image quality was in all cases rated as “without S-Enhance superior.”

### Influencing factors

**Fig. 7:**
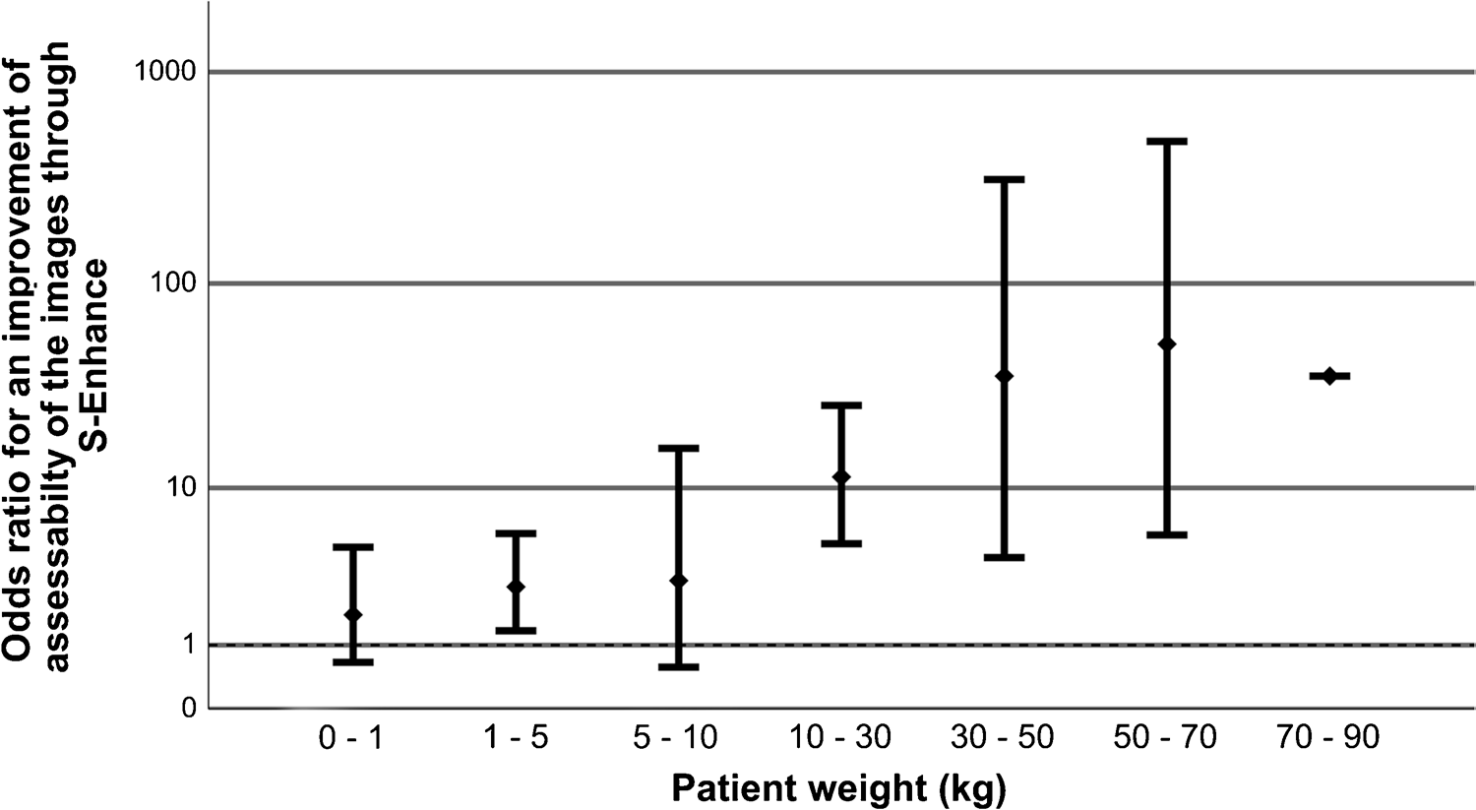
A bar chart shows the odds ratio (OR) for an improvement of interpretability of the images using S-Enhance influenced by patient weight, reference line for OR > 1 (improvement)

**Fig. 8:**
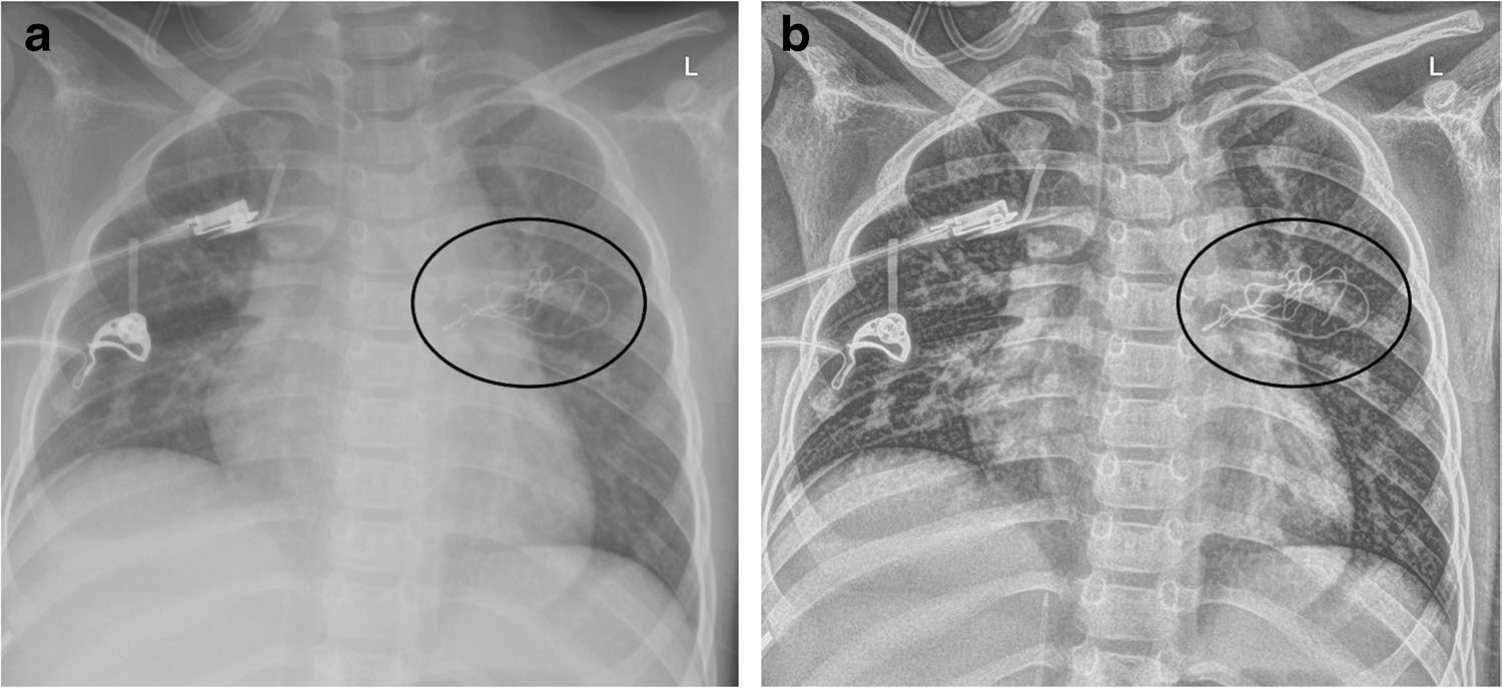
An anteroposterior chest radiograph (69.6 kV, 4.0 mAs, 3.91 cGy·cm^2^, exposure index: 376.78) unprocessed image (**a**) versus an S-Enhance image (**b**) in a 2-year-old girl (weight: 11 kg) with postinterventional foreign material (x-ray marker on compress) in projection to the left part of the chest (*black circle*)

**Fig. 9:**
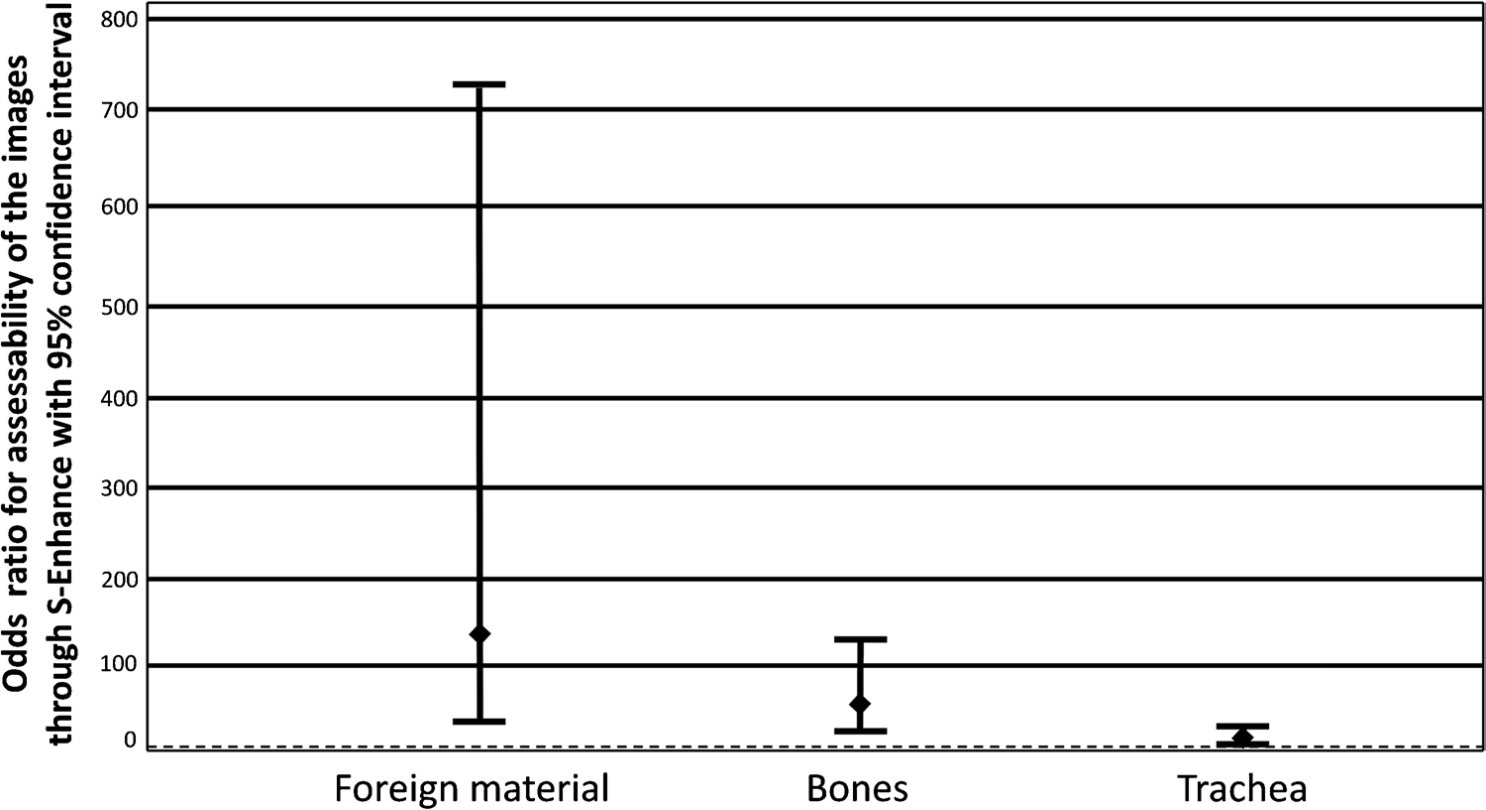
Results differentiate between different evaluation parameters for S-Enhance with a red line marking odds ratio (OR) = 1

With a differentiated view, S-Enhance showed significant image improvement for patients older than 6 months (OR for 0–6 month = 2.00, *P* = 0.02; OR 6 months to 1 year = 21.1, *P* < 0.05) and with a weight above 1 kg (OR < 1 kg = 1.778, *P* = 0.225, OR 1–2 kg = 3.24, *P* < 0.05) (Fig. 7). There was improvement especially for indwelling support devices (OR = 163.11, *P* < 0.05) and bone structures (OR = 34.92, *P* < 0.05) (Figs. 8 and 9). Results for all evaluated image parameters are demonstrated in Table 3. Furthermore, increasing image improvement was shown with rising exposure conditions (mAs, dose products) (Tables 4 and 5).

**Table 3:** Odds ratio (OR) for interpretability of the radiographs using S-Enhance for the different radiographic evaluation parameters

S-Enhance			95% confidence interval for OR		
Parameters	*n*	OR	Lower bound	Upper bound	*P*-value
Bones	76	34.92	9.70	125.69	**<0.0001**
Trachea	76	6.86	2.54	18.50	**<0.0001**
Central vessels	76	12.04	4.15	34.93	**<0.0001**
Peripheral vessels	76	0.30	0.12	0.78	**0.01**
Retrocardiac vessels	76	14.06	4.43	44.68	**<0.0001**
Foreign material	76	136.11	25.64	722.58	**<0.0001**
Quality regarding the clinical question	76	0.73	0.30	1.78	0.49

*n* number of evaluations

Bold indicates statistical significance (*P* < 0.05)

**Table 4:** Odds ratio (OR) for interpretability of the radiographs using S-Enhance for different current–time products

S-Enhance			95% confidence interval for OR		
Current–time product in mAs	*n*	OR	Lower bound	Upper bound	*P*-value
< 1	13	3.746	1.20	11.66	**0.02**
1–2	42	4.000	2.06	7.75	**<0.0001**
2–3	16	9.911	3.93	25.02	**<0.0001**
3–4	3	36.000	4.17	310.87	**0.001**
4–5	2	36.000	36.00	36.00	**<0.0001**

*n* number of evaluations

Bold indicates statistical significance (*P* < 0.05)

**Table 5:** Odds ratio (OR) for interpretability of the radiographs using S-Enhance for different dose products

S-Enhance			95% confidence interval for OR		
Dose product in cGy x cm^2^	*n*	OR	Lower bound	Upper bound	*P*-value
< 0,5	35	2.580	1.41	4.73	**0.002**
0,5–1	18	6.250	2.22	17.58	**0.001**
1–3	10	19.225	4.35	84.95	**<0.0001**
3–5	4	69.444	19.55	246.63	**<0.0001**
> 5	6	35.000	3.39	184.21	**0.002**

*n* number of evaluations

Bold indicates statistical significance (*P* < 0.05)

Both readers assessed the radiographs independent from each other at different time points. All evaluations were gathered and compared. When comparing the evaluations of both reviewers, there was no significant difference in the estimation of image quality with SimGrid or S-Enhance with an OR of 1.01. In general, both readers had a sixfold higher probability of a significant positive image evaluation using post-processing algorithms (OR of 6.55, *P* < 0.0001).

## Discussion

In clinical work with children and especially with neonates, x-ray imaging plays an important diagnostic role for clinically similar diseases, postoperative controls and position control of indwelling support devices [15, 16], but it can only be practiced with a strict indication as ionising radiation potentially harms human tissue [17]. Stochastic radiation damage is especially relevant, including long-term damage in cells causing cancer and damage to the genome [18]. Reducing patient dose while maintaining image quality is important (the ALARA [as low as reasonably achievable] principle) [19]. Beside guidelines [20], digital radiography and detector technologies, post-processing algorithms like SimGrid could potentially contribute to dose reduction. In the present study, we observed that SimGrid can improve image quality for all typical paediatric intensive care unit radiographs. Image quality improvement using post-processing increased with weight. The positive effect of SimGrid concerning image quality was manifested for all patients weighing more than 10 kg. SimGrid is designed to extract scattered radiation from x-ray images, which occurs very rarely in patients with low body mass (< 10 kg), meaning less thickness and smaller field sizes. Older children in general have a larger body mass (weight and thickness), causing more scattered radiation [21] that can be corrected by the software. Therefore, anti-scatter grids should only be used for older children [8] or according to newer insights for chest sagittal diameter greater than 12–15 cm [22]. Further, use of SimGrid was more effective for higher exposure conditions, which also relates to greater mass and body thickness of older patients causing more scattered radiation.

SimGrid was evaluated in a phantom study by Lee [5] and a clinical trial by Ahn et al. [7]. Both study groups compared SimGrid images with unprocessed non-SimGrid images as well as conventional grid images representative for adults only. In both studies SimGrid images were rated higher than the unprocessed images. Lee et al. [5] also showed that SimGrid images reached the same approximate quality as grid images. Similar results were demonstrated by Ahn et al. [7]. Their results differ from our results concerning the evaluation of lung structures, not improved in this work but seen by Ahn et al. [7], who also showed an 18.7% dose reduction using SimGrid. One reason for this might be the differing interaction from x-rays with the smaller body size of children resulting in less effectiveness of this post-processing algorithm.

To summarize, we have shown that SimGrid is a useful tool for image improvement in radiographs of children weighing more than 10 kg, but below that weight the effect is less pronounced and for newborns it has no effect.

Using S-Enhance, 61% of the radiographs were rated superior to the non-processed images while for 25% the overall image quality of the original image was preferred by the radiologists. When differentiating between the various parameters, S-Enhance significantly improves identification of foreign material, which is its main purpose. To our knowledge, there are no other published studies assessing the use of S-Enhance in the paediatric population. With respect to the overall quality, especially image noise impression, all S-Enhance x-ray images were rated lower than the original images. All in all, S-Enhance improves assessment of specific parameters such as bones and indwelling support devices for children, regardless of their weight but does not have positive effects on the overall image quality of x-rays. This makes it a helpful tool for specific questions but not for general diagnostics.

It is worth noting that we used radiographs obtained as part of the daily hospital routine, which does not make them fully comparable to standardized trials with phantoms but much more representative of clinical work.

The analysis of data in this clinical study could not be done under perfect conditions as there was not the same amount of data available for each group of patients. To avoid having the patients undergo an additional examination including another x-ray exposition, images obtained solely for clinical diagnosis were used. A second image of each patient would have been necessary to compare x-ray doses, which would cause higher radiation risks (especially for preterm infants). For this reason, we cannot make a statement concerning potential dose reduction using the algorithms, which is important. Another limitation is the different and, to some extent, small number of patients in each group, which might account for the large variations in the OR.

As previously mentioned, there are guidelines suggesting the use of anti-scatter grids for children older than 8 years [8]. There are also publications demonstrating that the sagittal diameter is a better factor [1] than patient weight and might be included in future guidelines [22]. As chest diameter of the patients was not available in our system, we grouped patients based on their weight. But for further studies it might be advisable to use sagittal chest diameter for analysis as it has the most informative value concerning scattered radiation.

## Conclusion

Using post-processing algorithms for radiography in neonatology and paediatric intensive care units improves the assessment of x-ray images in several ways: S-Enhance can be used in neonates to answer questions regarding the placement of indwelling support materials or bone pathologies, whereas SimGrid has a wide range of image improvement potential (overall quality, foreign material, bones, trachea, intestinal gas) for children weighing more than 10 kg, making it useful for the paediatric intensive care unit.
