# Correction to: Evaluation of the post‑processing algorithms SimGrid and S‑Enhance for paediatric intensive care patients and neonates

**DOI:** 10.1007/s00247-022-05353-3

**Published:** 2022-04-04

**Authors:** Paul‑Christian Krueger, Katharina Ebeling, Matthias Waginger, Katja Glutig, Marcel Scheithauer, Peter Schlattmann, Hans Proquitté, Hans‑Joachim Mentzel

**Affiliations:** 1 Section of Pediatric Radiology, Department of Diagnostic and Interventional Radiology, University Hospital Jena, Jena, Germany; 2 Department of Diagnostic and Interventional Radiology, University Hospital Jena, Jena, Germany; 3 Center for Clinical Studies, University Hospital Jena, Jena, Germany; 4 Section of Neonatology and Pediatric Intensive Care Medicine, Department of Pediatrics, University Hospital Jena, Jena, Germany

**Correction to: Pediatric Radiology (2022)**

**https://doi.org/10.1007/s00247-021-05279-2**

Figure 4 of the original published online version has incorrectly labelled subparts: Figure 4a as published online should be Figure 4b. Figure 4b as published online should be Figure 4a.

The original article has been corrected.

